# Appressoria-Producing Sordariomycetes Taxa Associated with *Jasminum* Species

**DOI:** 10.3390/pathogens12121407

**Published:** 2023-11-29

**Authors:** Deecksha Gomdola, Eric H. C. McKenzie, Kevin D. Hyde, Digvijayini Bundhun, Ruvishika S. Jayawardena

**Affiliations:** 1 School of Science, Mae Fah Luang University, Chiang Rai 57100, Thailand; deeckshagomdola@gmail.com (D.G.); kdhyde3@gmail.com (K.D.H.); 6371105502@lamduan.mfu.ac.th (D.B.); 2 Center of Excellence in Fungal Research, Mae Fah Luang University, Chiang Rai 57100, Thailand; 3 Manaaki Whenua-Landcare Research, Auckland 1072, New Zealand; mckenziee@landcareresearch.co.nz; 4 Innovative Institute for Plant Health, Zhongkai University of Agriculture and Engineering, Guangzhou 510225, China; 5 Kyung Hee University, Seoul 02447, Republic of Korea

**Keywords:** novel taxon, *Ciliochorella*, *Coniella*, infection pegs, phylogeny, poisson tree processes, *Pseudoplagiostoma*, taxonomy

## Abstract

Appressoria are specialized structures formed by certain phytopathogenic fungi during the early stages of the infection process. Over the years, significant advancements have been made in understanding the formation, types, and functions of appressoria. Besides being formed primarily by fungal pathogens, many studies have reported their occurrence in other life modes such as endophytes, epiphytes, and saprobes. In this study, we observed the formation of appressoria in fungal genera that have been found associated with leaf spots and, interestingly, by a saprobic species. We used morphological descriptions and illustrations, molecular phylogeny, coalescent-based Poisson tree processes (PTP) model, inter- and intra-species genetic distances based on their respective DNA markers, and Genealogical Concordance Phylogenetic Species Recognition Analysis (GCPSR) to establish a new species (*Pseudoplagiostoma jasmini*), a *Ciliochorella* sp., and a new host record (*Coniella malaysiana*). The *Ciliochorella* sp. is reported as a saprobe, while *Pseudoplagiostoma jasmini* and *Coniella malaysiana* were found to be associated with leaf spots of *Jasminum* species. All three taxa produce appressoria, and this is the first study that reports the formation of appressoria by a *Ciliochorella* sp. and a *Pseudoplagiostoma* sp.

## 1. Introduction

Appressoria are infection pegs, mostly produced by pathogenic fungi [1]. However, since these structures are also produced by endophytes, epiphytes, and saprobes, Chethana et al. [1] proposed a general definition of appressoria as “specialized cells or adhesion structures produced by fungi from which a penetration peg emerges that pierces or enters the host tissues”. Frank [2] discovered appressoria and came up with this term when he isolated the pathogen, *Colletotrichum lindemuthanium*, that causes diseases of beans. Based on the various shapes and sizes, appressoria can be grouped either as single-celled or multi-cellular/compound structures [3]. Single-celled appressoria are sub-divided into proto-appressoria, hyaline, and melanized appressoria. Compound appressoria are further classified as expressoria, infection cushion, and infection plaques [1,3].

Overall, in pathogenesis, appressoria are important for the successful invasion of host plants by certain pathogenic fungi. By attaching to the host, generating turgor pressure, and facilitating penetration, these structures ensure that the pathogen can overcome physical barriers and initiate infection of the plant [1,4]. The most frequently observed appressoria among several fungal species are single-celled, occurring mostly at the tip of germ tubes, sometimes formed laterally or intercalary on hyphae [1,3]. In this study, we identified three taxa isolated from *Jasminum* spp. that produce appressoria.

*Jasminum* (Oleaceae), native to tropical and warm temperate regions in Asia, Africa, and Europe, comprises around 200 species [5], several of which are also ecologically and economically important worldwide [6]. They are cultivated as ornamental plants, but they also have traditional and horticultural significance [7,8]. The leaves, stems, bark, roots, and flowers possess beneficial properties, including aphrodisiac, antiseptic, and diuretic [9]. Leaves of *J. grandiflorum* are used to cure odontalgia, otorrhea, otalgia, dysmenorrhea, leprosy, ulcerative stomatitis, ulcers, and wounds, among other disorders [10,11].

Several studies have reported fungi from *Jasminum* species. These studies include *Colletotrichum jasminigenum* and *C. siamense* on living leaves and flowers of *J. sambac* in Vietnam [12]; *Curvularia prasadii* isolated from leaves of *J. sambac* [13]; *Dothidea kunmingensis* reported from *J. nudiflorum* in southwestern China [14]; and *Puccinia aizazii*, a rust fungus, reported on *J. humile* from the foothills of the Himalayan ranges, Pakistan [15].

In this study, we employed a polyphasic approach to identify the three species, which resulted in one new taxon (*Pseudoplagiostoma jasmini*), a *Ciliochorella* sp., and a new host record (*Coniella malaysiana*). We used morphological descriptions and illustrations, molecular phylogeny, a coalescent-based Poisson tree processes (PTP) model, inter- and intra-species genetic distances based on their respective DNA markers, and Genealogical Concordance Phylogenetic Species Recognition Analysis (GCPSR). Updated phylogenetic trees comprising all species with molecular data are provided for the three genera. We also present drawings to show the variation in conidial shapes of *Pseudoplagiostoma* species. All three taxa belong to Sordariomycetes and were isolated from *Jasminum* spp. in northern Thailand. The *Ciliochorella* sp. is reported from dead leaves as a saprobe, while *Pseudoplagiostoma jasmini* and *Coniella malaysiana* were found to be associated with leaf spots. Single-celled hyaline appressoria were observed in these taxa. This is the first study that reports the formation of appressoria in *Ciliochorella* spp. and *Pseudoplagiostoma* spp.

## 2. Materials and Methods

### 2.1. Collection, Isolation and Morphological Analysis

Fallen leaf specimens with leaf spots and dead leaves of *Jasminum* spp. were collected from different sites in Chiang Mai, Thailand, in October 2019 and 2021, during the wet season. These were carried to the laboratory in paper bags. Single-spore isolation was performed as outlined by Senanayake et al. [16]. Axenic cultures were grown on malt extract agar (MEA, 50 g/L) and incubated for three to four weeks at 25 °C. Appressoria were observed after 24–48 h, forming at the tip of the germ tubes of the conidia. Free-hand sections of conidiomata were performed to examine the morphological characters of each species. Sterilized water was used as the mounting reagent to observe the different fungal features. A Motic SMZ 168 Series stereomicroscope was used to observe their morphology. Micro-morphological characters were photographed with a Canon 750D camera (Canon, Tokyo, Japan) attached to a Nikon ECLIPSE E600 compound microscope (Nikon, Tokyo, Japan). The photo-plates were assembled in Adobe Photoshop CS6 version 2020 (Adobe Systems Inc., San Jose, CA, USA), and measurements were made using Tarosoft^®^ Image Frame Work software (v.0.97).

### 2.2. Material Deposition and Reference Numbers

The holotype specimens and ex-type living cultures were deposited in the Mae Fah Luang University herbarium (MFLU) and Mae Fah Luang University Culture Collection (MFLUCC), respectively. FacesofFungi “https://www.facesoffungi.org/ (accessed on 20 November 2023)” and Index Fungorum numbers are given [17,18], with the species description updated in the GMS microfungi database “https://gmsmicrofungi.org/ (accessed on 20 November 2023)” [19]. Species identification and the establishment of the new taxon were based on Chethana et al. [20], Jayawardena et al. [21], and Maharachchikumbura et al. [22].

### 2.3. DNA Extraction, PCR Amplification, and Sequencing

Fresh mycelia, grown and incubated at 25 °C on MEA plates for four weeks, were scraped from the margins of colonies. Genomic DNA was extracted from these mycelia by using the Forensic DNA Kit (D3396-01, OMEGA bio-tek, Inc., Winooski, VT, USA), following the guidelines of the manufacturer. The loci of internal transcribed spacer (ITS, nuclear rDNA consisting of ITS1-5.8S-ITS2) and large subunit (28S, D1–D2 domains of nuclear 28S rDNA), and the genes for beta-tubulin (*β-tub*), RNA polymerase 2 (*Rpb2*), and translation elongation factor 1α (*tef-1α*) were amplified using the following primers: ITS5/ITS4 for ITS [23]; LR0R/LR5 for 28S [23]; Bt2a/Bt2b for *β-tub* [24]; Rpb2-5F2/7CR for *Rpb2* [25,26]; and EF1-728F/EF2 for *tef-1α* [27,28]. The polymerase chain reaction (PCR) was carried out in an Applied Biosystems C1000 TouchTM Thermal Cycler under the following conditions: Initial denaturation at 95 °C for 3 min; denaturation at 95 °C for 45 s; annealing at 55 °C for 50 s (ITS), 52 °C for 50 s (28S), 58 °C for 1 min 30 s (*β-tub*, *Rpb2*, and *tef-1α*); extension at 72 °C for 1 min; and final extension at 72 °C for 10 min (number of cycles = 40). The PCR mixture, totaling 25 µL, comprised 12.5 µL of Taq mix (PROMEGA GoTaq^®^, Green master mix, Madison, WI, USA), 1.5 µL of genomic DNA, 1 µL of the forward and reverse primer each, and 9 µL of double-distilled water.

The results of the amplification procedure were visualized using gel electrophoresis (1.7% agarose gel) by loading the resulting amplicons and DNA fluorescent loading dye (FluoroDye^TM^, SMOBIO, Seoul, Republic of Korea) in the sample wells. These amplicons were purified, and DNA was sequenced at SolGent Co. (Daejeon, Republic of Korea). Consensus sequences of the forward and reverse DNA sequence data were produced using SeqMan (DNAStar, Madison, WI, USA).

Accession numbers for all sequences deposited in the NCBI GenBank database “https://submit.ncbi.nlm.nih.gov/ (accessed on 20 November 2023)” are listed (Table 1).

### 2.4. Phylogenetic Analyses

A BLAST search in NCBI “https://blast.ncbi.nlm.nih.gov/ (accessed on 20 November 2023)” was conducted for our sequences, and sequence data of ITS, 28S, *β-tub*, *Rpb2*, and *tef-1α* from related species were retrieved from GenBank (https://www.ncbi.nlm.nih.gov/ accessed on 20 November 2023) (Table 1). Sequences were aligned using MAFFT v.7 by applying the default settings (https://mafft.cbrc.jp/alignment/server/ accessed on 20 November 2023) [29] and trimmed using trimAl [30]. Individual loci were combined using BioEdit v.7.0.5.2 [31]. Phylogenetic trees were constructed using maximum likelihood (ML), maximum parsimony (MP), and Bayesian inference (BI) methods. Both single and combined gene trees were constructed to compare the topology and taxonomic placement of each taxon.

Maximum likelihood analyses (ML-IQ) were performed in the webserver (https://iqtree.cibiv.univie.ac.at/ accessed on 20 November 2023), by selecting the default parameters and 1000 ultrafast bootstrap replicates [32]. Phylogenetic Analysis Using Parsimony (PAUP) v.4.0b10 was used to compute MP analyses [33]. A heuristic search option with the addition of 1000 random sequence additions was applied. Maxtrees and bootstrap replicates were set up to 1000. Bayesian inference analysis (MrBayes on XSEDE v.3.2.7a) was performed in the CIPRES Science Gateway v.3.3 [34,35], after implementing MrModeltest to estimate the model of evolution of individual gene regions [36]. The partition model for each gene region is given (Table 2). Markov chain Monte Carlo (MCMC) sampling with four Markov chains was used to infer posterior probabilities (PP) for 1,000,000, 5,000,000, and 2,000,000 generations for *Ciliochorella*, *Coniella*, and *Pseudoplagiostoma*, respectively. The tree sample frequencies were set to 100. The first 20% of the total trees were discarded as “burn in” and the remaining 80% was used to calculate posterior probabilities.

FigTree v.1.4.4 was used to display the phylogenetic trees [37], and the phylograms were edited and produced in Microsoft PowerPoint (2016).

### 2.5. Genealogical Concordance Phylogenetic Species Recognition Analysis (GCPSR)

The GCPSR model was applied to scrutinize any significant recombination event that occurred between the new taxon and other phylogenetically closely related species [38], as inferred by a pairwise homoplasy index (Φw) (PHI) test. The analysis was performed in SplitsTree4 by applying the LogDet transformation and splits decomposition options [39,40]. The final layout of the splitsTree graphs was produced in Microsoft PowerPoint (2016).

### 2.6. Poisson Tree Processes (PTP)

The coalescent-based PTP model was applied to delineate species in *Pseudoplagiostoma*. The analysis was computed on the Web Server (https://species.h-its.org/ptp/ accessed on 20 November 2023) [41]. The model assumes that the process of speciation is marked by a branching event in the evolutionary tree of a group of organisms, which separates the ancestral lineage into two or more new lineages. The model further assumes that the number of lineages in a group evolves according to a Poisson process, with the rate of speciation being proportional to the number of lineages. The PTP analysis was based on the concatenated ITS, 28S, *β-tub*, and *tef-1α* regions. Maximum likelihood analysis prior to computing PTP was conducted on the IQ-tree Web Server. Genetic distances were calculated in MEGA-X by applying the Kimura 2-parameter substitution model and selecting the gamma distribution and pairwise deletion options.

## 3. Results

### 3.1. Sequence Alignment and Phylogenetic Analyses

The number of strains used in the phylogenetic analyses of each genus is given (Table 3). Phylogenetic analyses from single and combined gene regions support the identification of the new species (*Pseudoplagiostoma jasmini*), a *Ciliochorella* sp., and the new host record, *Coniella malaysiana*. The phylogenetic trees generated from ML-IQ, MP, and BI yielded similar taxonomic placements for our isolates.

The *tef-1α* of *Pseudoplagiostoma mangiferae* was excluded from the phylogenetic analyses because when we used the BLAST tool for *P. mangiferae* (accession number: MK084822; 100% identity; 100% query cover; e-value = 0.0), the sequence tallied with *Melanconis* instead of *Pseudoplagiostoma*.

### 3.2. Analysis 1: Ciliochorella 

**Figure 1:**
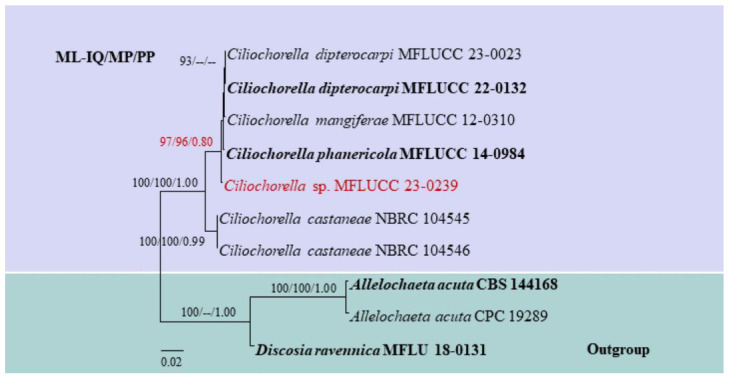
Maximum likelihood phylogram based on the combined ITS, 28S, and *β-tub* matrices of *Ciliochorella*. Bootstrap support values (ML-IQ ≥ 80%), maximum parsimony (MP ≥ 80%), and Bayesian posterior probabilities (PP ≥ 0.80) are given above the branches or at the nodes as ML-IQ/MP/PP. Hyphen (-) indicates bootstrap support values below 80% for ML-IQ and MP, and posterior probabilities below 0.80. *Allelochaeta acuta* (CBS 144168 and CPC 19289) and *Discosia ravennica* (MFLU 18-0131) are the outgroup taxa. Ex-type and reference strains are in bold, and our isolate is in red.

Based on the combined ITS, 28S, and *β-tub* sequence data of *Ciliochorella*, our isolate, MFLUCC 23-0239, clusters with other *Ciliochorella* species and forms a distinct lineage with the larger subclade in which reside *C. dipterocarpi*, *C. mangiferae*, and *C. phanericola* (97% ML-IQ, 96% MP, 0.88 PP) (Figure 1).

#### Genealogical Concordance Phylogenetic Species Recognition Analysis (GCPSR)

**Figure 2:**
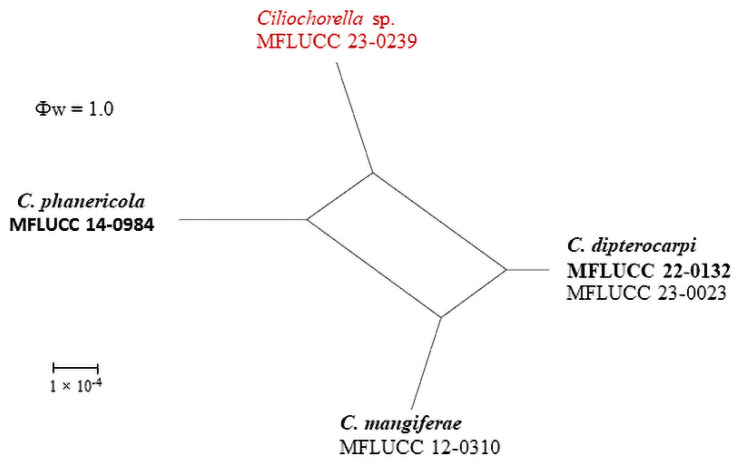
Split graph derived from the PHI analysis, generated for *Ciliochorella*. Our isolate is in red.

The LogDet transformation and splits decomposition options were selected while configuring the PHI test. The analysis yielded a threshold over 0.05 (Φw = 1.0) for the *Ciliochorella* sp., indicating no significant recombination event (Figure 2).

### 3.3. Analysis 2: Coniella

**Figure 3:**
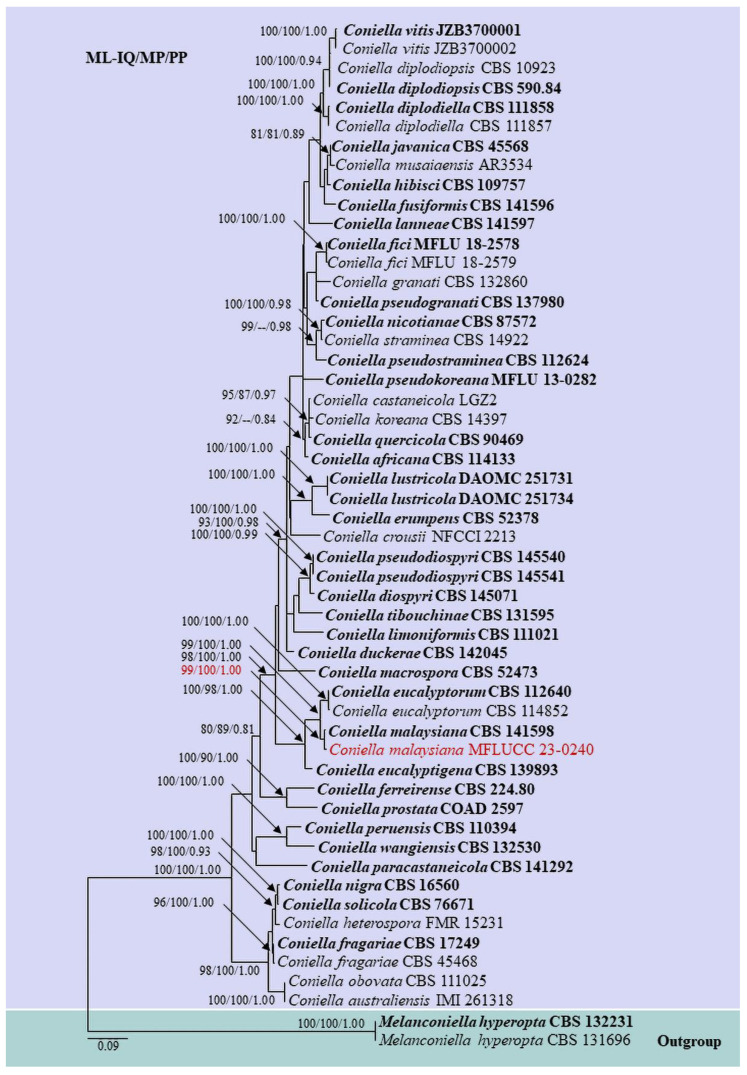
Maximum likelihood phylogram based on the combined ITS, 28S, *Rpb2*, and *tef-1α* matrices of *Coniella*. Bootstrap support values (ML-IQ ≥ 80%) and maximum parsimony (MP ≥ 80%), and Bayesian posterior probabilities (PP ≥ 0.80) are given above the branches or at the nodes as ML-IQ/MP/PP. Hyphen (-) indicates bootstrap support values below 80% for ML-IQ and MP, and posterior probabilities below 0.80. *Melanconiella hyperopta* (CBS 132231 and CBS 131696) are selected as outgroups. Ex-type and reference strains are in bold, and our isolate is in red.

Based on the combined ITS, 28S, *Rpb2*, and *tef-1α* sequence data of *Coniella*, our isolate, MFLUCC 23-0240, forms a sister clade with the ex-type of *C. malaysiana* with 99% ML-IQ and 100% MP bootstrap support, and 1.00 PP support (Figure 3).

### 3.4. Analysis 3: Pseudoplagiostoma

**Figure 4:**
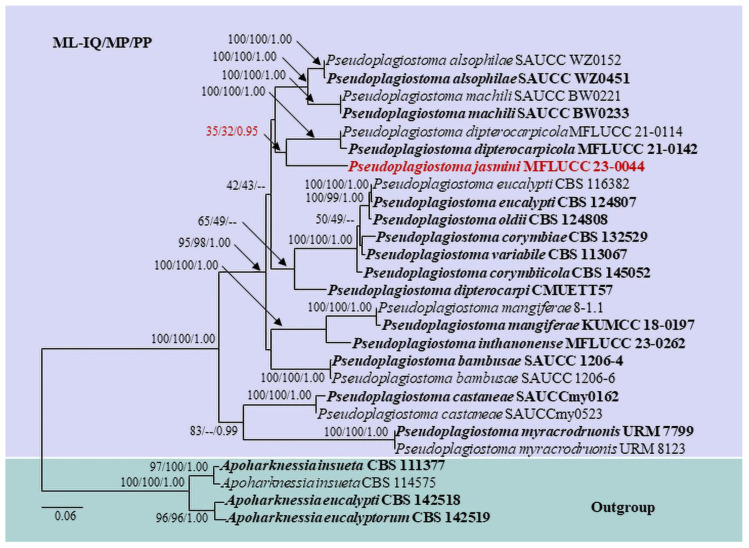
Maximum likelihood phylogram based on the combined ITS, 28S, *β-tub*, *Rpb2*, and *tef-1α* matrices of *Pseudoplagiostoma*. Bootstrap support values (ML-IQ ≥ 30%) and maximum parsimony (MP ≥ 30%), and Bayesian posterior probabilities (PP ≥ 0.80) are given above the branches or at the nodes as ML-IQ/MP/PP. Hyphen (-) indicates bootstrap support values below 30% for ML-IQ and MP, and posterior probabilities below 0.80. *Apoharknessia eucalypti* (CBS 142518), *A. eucalyptorum* (CBS 142519), and *Apoharknessia insueta* (CBS 111377 and CBS 114575) are the outgroup taxa. Ex-type and reference strains are in bold, and the new taxon is in bold red.

Based on the combined ITS, 28S, *β-tub*, *Rpb2*, and *tef-1α* sequences of *Pseudoplagiostoma*, our isolate, MFLUCC 23-0044, groups with other species of *Pseudoplagiostoma* and forms a sister clade with *P. dipterocarpicola* (MFLUCC 21-0142 and MFLUCC 21-0114) with 35% ML-IQ and 32% MP bootstrap support, and 0.95 PP support (Figure 4).

#### 3.4.1. Genealogical Concordance Phylogenetic Species Recognition Analysis (GCPSR)

**Figure 5:**
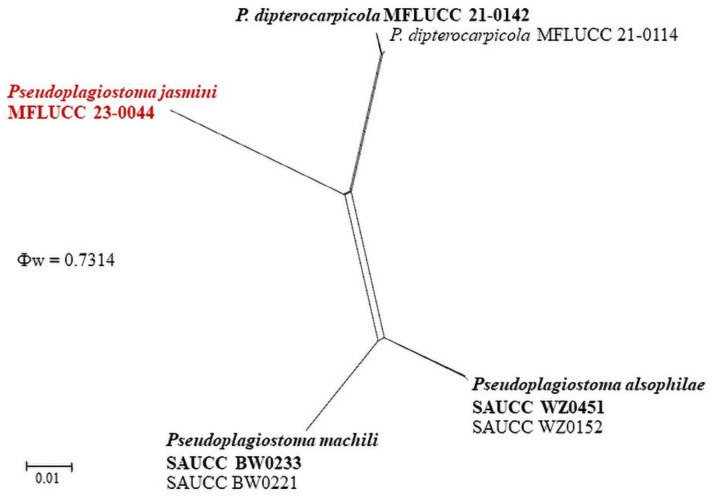
Split graph derived from the PHI analysis, generated for *Pseudoplagiostoma*. The novel species is in bold red.

The LogDet transformation and splits decomposition options were selected while configuring the PHI test. The analysis yielded a threshold over 0.05 (Φw = 0.7314) for the new species, *Pseudoplagiostoma jasmini*, indicating no significant recombination (Figure 5).

#### 3.4.2. Poisson Tree Processes

**Figure 6:**
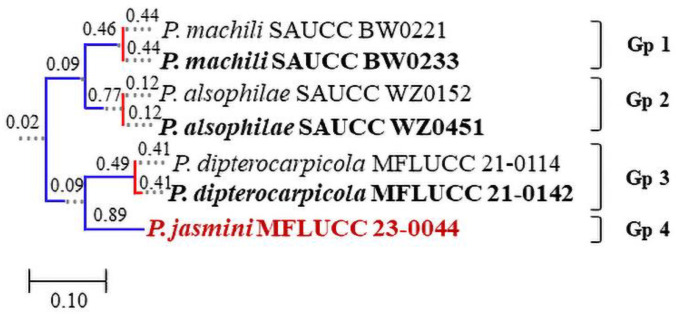
Results generated from the PTP analysis of *Pseudoplagiostoma*. The analysis was based on the ML-IQ topologies of the concatenated ITS, 28S, *β-tub*, and *tef-1α* matrices. Groups of species are denoted by colored branches, with blue-colored branches indicating that they are different species, and red-colored branches representing different strains of the same species. Numbers near the nodes are posterior probabilities. The new taxon is given in bold red.

The result generated from the PTP analysis (Figure 6) is congruent with the maximum likelihood phylogram that delimits *Pseudoplagiostoma jasmini* as a new species (Figure 4). Genetic distances of *Pseudoplagiostoma jasmini* compared with its phylogenetically closely related taxa are summarized in the “note” section under *Pseudoplagiostoma* in the “Taxonomy” section.

## 4. Taxonomy

### 4.1. Sporocadaceae Corda [as “Sporocadeae”], Icon. Fung. (Prague) 5: 34 (1842)

This family comprises saprobic, pathogenic, as well as endophytic genera that are commonly characterized by conidia that have appendages at one or both ends. Sporocadaceae has previously been subjected to multiple taxonomic re-evaluations and classifications [42,43]. Bartaliniaceae, Discosiaceae, Pestalotiopsidaceae, and Robillardaceae were previously treated as synonyms of Sporocadaceae [43,44,45].

#### 4.1.1. *Ciliochorella* Syd., in Sydow & Mitter, Annls Mycol. 33(1/2): 62 (1935)

Type species—*Ciliochorella mangiferae* Syd.

*Ciliochorella* (Sporocadaceae, Amphisphaeriales, Xylariomycetidae) [42,43,46,47] was established by Sydow and Mitter [48]. There are ten species in Index Fungorum [18] and nine species in Species Fungorum [49]. Among these, only five *Ciliochorella* species have sequence data for one or more gene loci. *Ciliochorella* species comprise saprobic taxa that have been reported from India, Japan, South America, and Thailand [42,50,51,52]. Our isolate is also reported in its saprobic mode.

The genus is characterized by cylindrical, straight, or slightly curved conidia that are eu-septate, usually bearing two to three or more tubular apical appendages and a single basal appendage.

#### 4.1.2. *Ciliochorella* sp. Gomdola, K.D. Hyde & Jayaward.

Saprobic on the leaves of *Jasminum* sp. Sexual morph: Not observed. Asexual morph: Coelomycetous. *Conidiomata* in cross-section 1000–1100 μm diam., 370–380 μm high ($\bar{x}$ = 1042 × 373 μm, n = 5), acervulus, semi-immersed, carbonaceous, solitary, uniloculate, black. *Conidiomata wall* 40–53 μm diam. ($\bar{x}$ = 46.7 μm, n = 10), consisting of several layers of pseudoparenchymatous cells of *textura angularis*, outer layers dark brown, inner layers pale brown to hyaline. *Conidiophores* indistinct, often reduced to conidiogenous cells. *Conidiogenous* cells phialidic, (5.2–)6.7–8.5(–9.5) × 1.9–2.8 μm ($\bar{x}$ = 7.4 × 2.4 μm, n = 10), formed from the inner-most layer of the wall, hyaline to pale brown, ampulliform, smooth-walled, proliferating enteroblastically. *Conidia* 11–15 × 2.4–3.8 μm ($\bar{x}$ = 12.9 × 3.3 μm, n = 50) (excluding basal cell), hyaline to pale brown, guttulate, 1-euseptate, smooth-walled, allantoid to sub-cylindrical, or sub-falcate to reniform, apex sometimes broadly obtuse, tapering towards a slightly curved base with a hyaline obconic *basal cell* 2.8–4.5 μm long ($\bar{x}$ = 3.6 μm, n = 30); conidia bearing 2 apical and 1 basal appendage. *Appendages* tubular, filiform, flexuous, *apical appendages* (6.5–)12.5–18.5 μm long ($\bar{x}$ = 16.5 μm, n = 50), *basal appendage* (2.5–)4–6.5(–8) μm long ($\bar{x}$ = 5.4 μm, n = 50). *Appressorium* 20 × 18.5 μm, single-celled, cordate to irregular-shaped, hyaline.

Culture characteristics: Colonies on MEA reaching approximately 20 mm diam. after 14 days of incubation at 25 °C, elevation flat, forming concentric rings with an entire margin, mycelium white.

Material examined: Thailand, Chiang Mai Province, Doi Lo district, on fallen dead leaves of *Jasminum* sp. (Oleaceae), 15 October 2019, D. Gomdola, DG314 (MFLU 23-0388), living culture MFLUCC 23-0239.

GenBank accession numbers: ITS = OR610581, 28S = OR610582.

Notes: *Ciliochorella* sp. (MFLUCC 23-0239) groups with other *Ciliochorella* species and forms a separate lineage with the larger subclade in which reside *C. dipterocarpi*, *C. mangiferae*, and *C. phanericola* (97% ML-IQ, 96% MP, 0.88 PP) (Figure 1). The conidial features match the morphological species concept of *Ciliochorella*. We compared the morphology of *Ciliochorella* sp. (MFLUCC 23-0239) with that of its phylogenetically closely related, *C. phanericola*. The conidial shape, color, and size of *Ciliochorella* sp. (MFLUCC 23-0239) and *C. phanericola* are mostly similar (Table 4). However, the conidia of *Ciliochorella* sp. (MFLUCC 23-0239) are 1-euseptate, while those of *C. phanericola* are 2-septate. Both the apical and basal appendages of *Ciliochorella* sp. (MFLUCC 23-0239) are shorter than those of *C. phanericola* (Table 4). The growth rate of *Ciliochorella* sp. (MFLUCC 23-0239) (2 cm after 14 days) is slower than that of *C. phanericola* (2.5 cm after 7 days), both grown on MEA and incubated at 25 °C [51]. In addition, appressoria were not observed in *C. phanericola* [51].

Excluding gaps in our aligned untrimmed dataset, in comparison of the inter-species genetic distance of *Ciliochorella* sp. (MFLUCC 23-0239) and *C. phanericola*, a difference of 0.34% was seen across ITS (533 nucleotides), but no difference was observed across 28S (868 nucleotides). We were unable to compare the differences across *β-tub* as *Ciliochorella* sp. (MFLUCC 23-0239) lacks sequence data for the gene region. Despite several trials using different amplification conditions, we were unable to obtain sequence data for *β-tub*. Therefore, coupled with morphological description and multi-locus phylogenetic analyses, a PHI test was also conducted to support the taxonomic placement of our isolate (MFLUCC 23-0239). The PHI test of the combined ITS and 28S yielded a threshold exceeding 0.05 (Φw = 1.0), suggesting that no recombination event has occurred.

**Figure 7:**
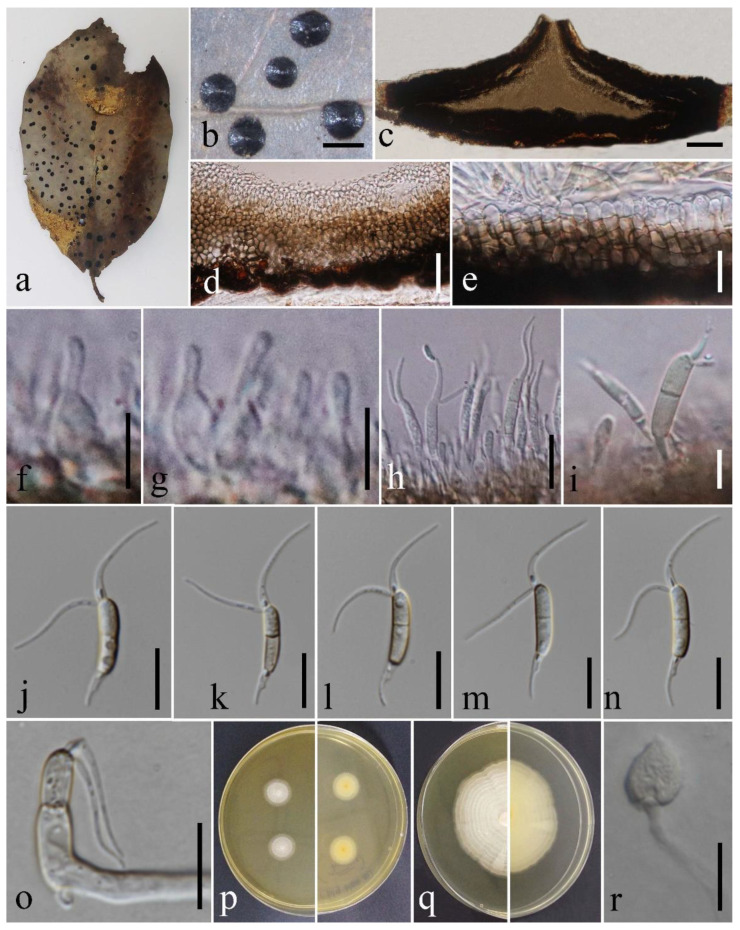
*Ciliochorella* sp. (MFLUCC 23-0239) (**a**) Leaf specimen. (**b**) Close up of conidiomata on a leaf of *Jasminum* sp. (**c**) Section through conidioma. (**d**,**e**) Conidiomata wall. (**f**–**i**) Conidiophores, conidiogenous cells and developing conidia. (**j**–**n**) Immature and mature conidia with appendages. (**o**) Germinated conidium (**p**,**q**) top (left) and reverse (right) of colonies on MEA after 7 and 14 days of incubation, respectively. (**r**) Appressorium. Scale bars: (**b**) = 1 mm, (**c**) = 100 μm, (**e**,**h**,**j**–**o**) = 10 μm, (**d**,**r**) = 20 μm, (**f**,**g**,**i**) = 5 μm.

Nevertheless, despite the PHI test result, we suggest establishing our isolate as *Ciliochorella* sp. instead of identifying it as a new species due to the lack of sequence data. Further studies focusing on the collection of more *Ciliochorella* taxa and providing sequence data for protein-coding gene regions (*β-tub*, *Rpb2*, *tef-1α*) will yield better resolution in the phylogenetic trees and contribute to proper species identification (Figure 7).

### 4.2. Schizoparmaceae Rossman, D.F. Farr & Castl. [as “Schizoparmeaceae”], Mycoscience 48(3): 137 (2007)

Schizoparmaceae was introduced to accommodate *Schizoparme* (sexual morph reported), *Coniella*, and *Pilidiella* (asexual morph reported) [53,54]. Alvarez et al. [55] revised the family and synonymized *Pilidiella* and *Schizoparme* under *Coniella*. Species in this family occur in tropical and temperate areas as phytopathogens as well as saprobes and endophytes [43,56].

#### 4.2.1. *Coniella* Höhn., Ber. Dt. Bot. Ges. 36(7): 316 (1918)

Type species—*Coniella pulchella* Höhn.

*Coniella* (Schizoparmaceae, Diaporthales, and Diaporthomycetidae) [43,46,47] was established by Höhnel [57]. There are 64 species in Index Fungorum [18] and 58 species in Species Fungorum [49]. Of these, 42 *Coniella* species have sequence data for one or more gene regions. The genus is primarily characterized by erumpent, brown to black ascomata or conidiomata, and hyaline conidia that become pigmented upon maturation [58,59].

#### 4.2.2. *Coniella malaysiana* L.V. Alvarez & Crous, in Alvarez, Groenewald & Crous, Stud. Mycol. 85: 21 (2016)

Index Fungorum number: IF 817823, Facesoffungi number: FoF 14882

Associated with leaf spots of *Jasminum* sp. Leaf spots irregular or oval to elongated, brown, surrounded by a dark brown to black margin, outermost surrounding reddish brown. Sexual morph: Not observed. Asexual morph: Coelomycetous. *Conidiomata* 135–140 μm diam., 100–130 μm high ($\bar{x}$ = 139 × 114 μm, n = 5), pycnidial, semi-immersed, sometimes erumpent, solitary, scattered or gregarious, uniloculate, globose to subglobose, black. *Conidiomata wall* 13.5–24.5(–28) μm diam. ($\bar{x}$ = 18.6 μm, n = 10), consisting of 3–4 layers of thick-walled pseudoparenchymatous cells of *textura angularis*, outer layers dark brown, inner layer pale brown to hyaline. *Conidiophores* 6.9–15 μm long ($\bar{x}$ = 11.2 μm, n = 10), straight to flexuous, cylindrical to ampulliform or oblong, hyaline, aseptate, unbranched, sometimes reduced to conidiogenous cells. *Conidiogenous cells* enteroblastic, phialidic, 6.9–13 × 2.1–3.4 μm ($\bar{x}$ = 10.7 × 2.6 μm, n = 10), hyaline, cylindrical or ampulliform, guttulate, smooth-walled. *Conidia* (7.5–)8.2–13.1 × 4–5 μm ($\bar{x}$ = 10.6 × 4.1 μm, n = 50), hyaline when immature, becoming pale to dark brown upon maturation, guttulate, aseptate, smooth, thick-walled, 0.4–1.5 μm diam. ($\bar{x}$ = 0.75 μm, n = 30), fusiform to truncate to sub-ellipsoidal, sometimes obovoid, wider in the middle, tapering towards a slightly curved apex and base, often with a prominent protruding basal *hilum*. *Appressoria* 19–23 × 9–15 μm ($\bar{x}$ = 20.9 × 12 μm, n = 2), single-celled, sub-ellipsoidal to irregular-shaped, hyaline.

Culture characteristics: Colonies on MEA reaching approximately 20 mm diam. after 7 days of incubation at 25 °C, elevation flat or raised, round with raised margin, forming concentric rings, mycelium dense and aerial, white.

Material examined: Thailand, Chiang Mai Province, Omkoi district, Yang Piang sub-district, associated with leaf spots of *Jasminum* sp. (Oleaceae), 16 October 2019, D. Gomdola, DG392 (MFLU 23-0389), living culture MFLUCC 23-0240.

Hosts and Distribution: Leaves of *Corymbia torelliana* in Malaysia [55], leaves of *Jasminum* sp. in Thailand (this study).

GenBank accession numbers: ITS = OR608286, 28S = OR608334, *Rpb2* = OR601568 and *tef-1α* = OR601569.

Notes: Our collection shares similar morphological characters with those of the ex-type, *C. malaysiana* (CBS 141598) [55]. Our strain and *C. malaysiana* (CBS 141598) have hyaline to brown, aseptate conidia with guttules [55]. Conidial sizes are mostly similar (Table 5). The conidial length-to-width ratio of our isolate is 2.6, and that of *C. malaysiana* (CBS 141598) is 2.5. Other morphological similarities and differences between the two strains of *C. malaysiana* are given (Table 5).

In the phylogenetic analyses of the combined ITS, 28S, *Rpb2*, and *tef-1α*, our isolate is sister to the ex-type of *C. malaysiana* (99% ML-IQ, 100% MP, 1.00 PP) (Figure 3). Excluding gaps in our aligned untrimmed dataset, upon comparison of the intra-species genetic distance between our strain and the ex-type of *C. malaysiana*, the following differences were observed: 0.55% across ITS (553 nucleotide base pairs, bp), 0.12% across 28S (827 bp), 0.26% across *Rpb2* (767 bp), but 2.4% across *tef-1α* (295 bp).

**Figure 8:**
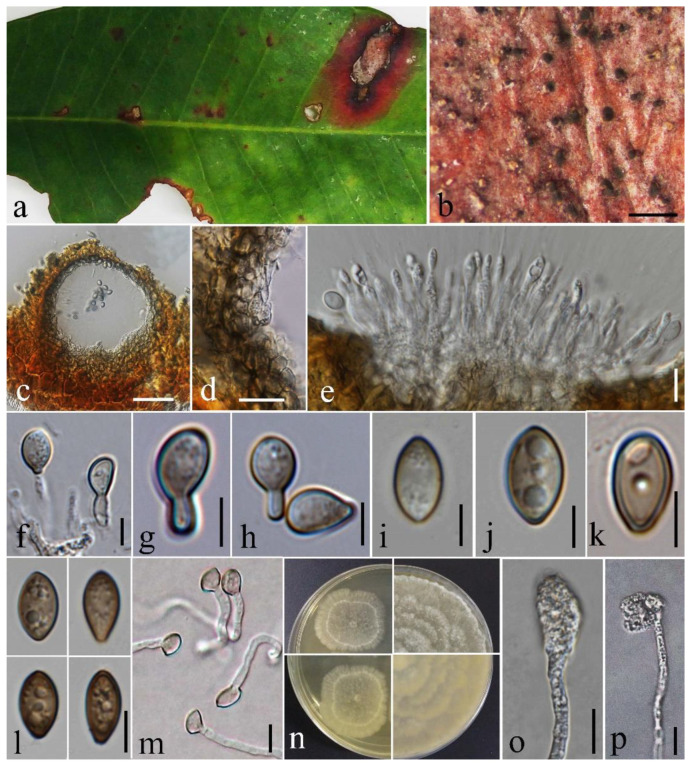
*Coniella malaysiana* (MFLUCC 23-0240) (**a**) Herbarium specimen with leaf spots. (**b**) Close up of conidiomata on a leaf of *Jasminum* sp. (**c**) Section through a conidioma. (**d**) Conidioma wall. (**e**–**h**) Conidiophores, conidiogenous cells, and developing conidia. (**i**–**l**) Immature and mature conidia. (**m**) Germinated conidia (**n**) Top (upper) and reverse (lower) of colony on MEA after 5 and 14 days of incubation. (**o**,**p**) Appressoria. Scale bars: (**b**) = 500 μm, (**c**) = 50 μm, (**d**,**e**,**m**,**o**,**p**) = 10 μm, (**f**–**l**) = 5 μm.

Based on morphology and multigene phylogenetic analyses, we identify our strain as a new host record of *Coniella malaysiana*, associated with leaf spots of *Jasminum* sp. in northern Thailand (Figure 8).

### 4.3. Pseudoplagiostomataceae Cheew., M.J. Wingf. & Crous [as “Pseudoplagiostomaceae”], in Cheewangkoon et al., Fungal Diversity 44: 95 (2010)

Pseudoplagiostomataceae, a monotypic family, was introduced to accommodate *Pseudoplagiostoma*, a genus that is morphologically similar but phylogenetically distinct to *Plagiostoma* [60].

#### 4.3.1. *Pseudoplagiostoma* Cheew., M.J. Wingf. & Crous, in Cheewangkoon et al., Fungal Diversity 44: 96 (2010)

Type species—*Pseudoplagiostoma eucalypti* Cheewangkoon, M.J. Wingf. & Crous

**Figure 9:**
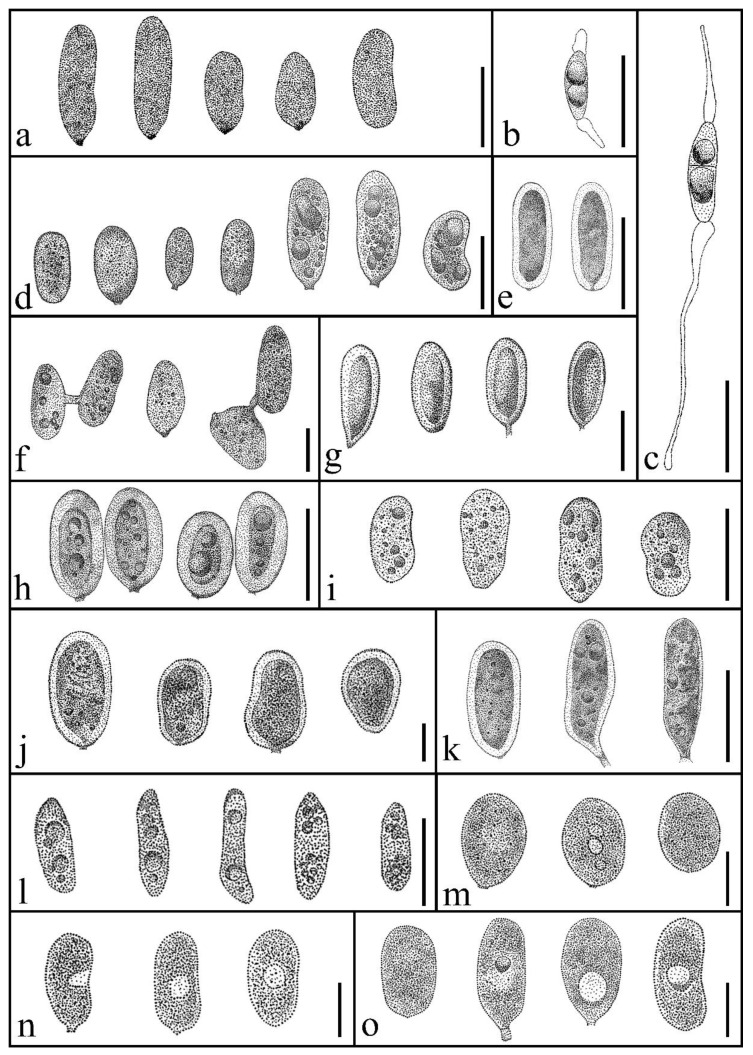
Conidial (**a**,**d**–**o**) and ascospores (**b**,**c**) morphology of *Pseudoplagiostoma* spp. (**a**) *P. eucalypti* (asexual morph) (**b**,**c**) *P. eucalypti* (ascospores with apical and basal appendages) (**d**) *P. oldii* (**e**) *P. dipterocarpi* (**f**) *P. variabile* (**g**) *P. corymbiicola* (**h**) *P. corymbiae* (**i**) *P. myracrodruonis* (**j**) *P. mangiferae* (**k**) *P. dipterocarpicola* (**l**) *P. castaneae* (**m**) *P. alsophilae* (**n**) *P. bambusae* (**o**) *P. machili*. Scale bars = 15 µm. (Redrawn from Cheewangkoon et al. [60]; Crous et al. [61,62]; Suwannarach et al. [63]; Bezerra et al. [64]; Phookamsak et al. [65]; Mu et al. [66]; Tang et al. [67]; Zhang et al. [68]).

*Pseudoplagiostoma* (Pseudoplagiostomataceae, Diaporthales, and Diaporthomycetidae) was established by Cheewangkoon et al. [60], with the introduction of three species: *P. eucalypti*, *P. oldii*, and *P. variabile*. There are 13 species in Index Fungorum [18] and nine species in Species Fungorum [49], and all 13 species have sequence data for one or more gene loci. The nomenclature of *Pseudoplagiostoma* reflects the morphological similarities with *Plagiostoma* (Gnomoniaceae, Diaporthales). Pseudoplagiostoma species have both sexual and asexual morphs [60]. Their sexual morph is characterized by perithecial, immersed, globose or elliptical ascomata, subcylindrical unitunicate asci (J-), and hyaline, ellipsoidal, and elongated ascospores, usually with a median septum and hyaline appendages at the apex and base (Figure 9). Their asexual morph consists of acervular or pycnidial conidiomata, and hyaline, smooth-walled, aseptate conidia (Figure 9) [60].

#### 4.3.2. *Pseudoplagiostoma jasmini* Gomdola, K.D. Hyde & Jayaward., sp. nov.

Index Fungorum number: IF 900131, Facesoffungi number: FoF 14104

Etymology: The specific epithet refers to the host genus, *Jasminum*, from which the species was isolated.

Holotype: MFLU 23-0068

Associated with leaf spots of *Jasminum grandiflorum*. Leaf spots irregular, pale to medium brown, surrounded by a dark brown to black margin. Sexual morph: Not observed. Asexual morph: Coelomycetous. *Conidiomata* (145–)150–230(–240) μm diam., (135–)140–200 μm high ($\bar{x}$ = 184 × 171 μm, n = 20), pycnidial, semi-immersed, solitary, scattered, uniloculate, globose to subglobose, pale brown, surrounded with black margin. *Conidiomata wall* (19–)22–42(–46) μm thick ($\bar{x}$ = 28 μm, n = 15), consisting of 3–4 layers of thick-walled pseudoparenchymatous cells of *textura angularis*, outer layers dark brown to black, inner layers pale brown to hyaline. *Conidiophores* indistinct, often reduced to conidiogenous cells. *Conidiogenous cells* phialidic, (6.8–)7.7–13.7(–15.6) × 1.6–2.4(–3.0) μm ($\bar{x}$ = 10.7 × 2.1 μm, n = 10), hyaline, cylindrical or clavate, guttulate, smooth-walled, proliferating enteroblastically. *Conidia* (11.8–)14–22 × (5.2–)6.5–11 μm (($\bar{x}$ = 18.5 × 9.5 μm, n = 50), hyaline, guttulate, 0–2-septate, smooth, wall (0.5–)0.6–1.3 μm thick ($\bar{x}$ = 0.8 μm, n = 50), cylindrical to truncate or ellipsoidal, elongated, reniform, pyriform or obovoid, apex broadly obtuse, tapering towards a slightly curved base, often with a prominent protruding *hilum*. *Appressorium* 9.6 × 7.2 μm long, single-celled, sub-ellipsoidal to obovoid or subglobose or irregular-shaped, hyaline.

Culture characteristics: Colonies on MEA reaching approximately 20 mm diam. after 14 days of incubation at 25 °C, immature with white mycelium, elevation flat or raised, becoming aerial dense and olivaceous brown, filamentous with an undulate margin when aged.

Material examined: Thailand, Chiang Mai Province, Doi Inthanon National Park, Kew Mae Pan nature trail, on fallen leaves of *Jasminum grandiflorum* (Oleaceae), 20 October 2021, D. Gomdola, DG-PSEU (MFLU 23-0068, holotype), ex-type living culture MFLUCC 23-0044.

GenBank accession numbers: ITS = OQ786078, 28S = OQ786079, *β-tub* = OQ850148 and *tef-1α* = OQ850145.

Notes: *Pseudoplagiostoma jasmini* groups with other species of *Pseudoplagiostoma* and forms a sister clade with *P. dipterocarpicola* (MFLUCC 21-0142 and MFLUCC 21-0114) with 35% ML-IQ, 32% MP, and 0.95 PP support (Figure 4). The features are congruent with the morphological species concept of *Pseudoplagiostoma* [60].

*Pseudoplagiostoma jasmini* varies substantially in conidial shape (Figure 9). Conidia of *P. jasmini* are longer than those of *P. dipterocarpicola* (MFLUCC 21-0142) (Table 6). The conidial length-to-width ratio of *P. jasmini* is 2.0, whereas that of *P. dipterocarpicola* is 2.7.

Excluding gaps in our aligned untrimmed dataset, in pairwise nucleotide comparisons of *P. jasmini* and *P. dipterocarpicola* (MFLUCC 21-0142), the following differences were observed: 5.76% across ITS (543 nucleotide base pairs, bp), 1.86% across 28S (818 bp), 21.1% across *β-tub* (448 bp), and 43.7% across *tef-1α* (164 bp). The inter-species genetic distances (%) grouped according to the PTP result are provided (Table 7).

**Figure 10:**
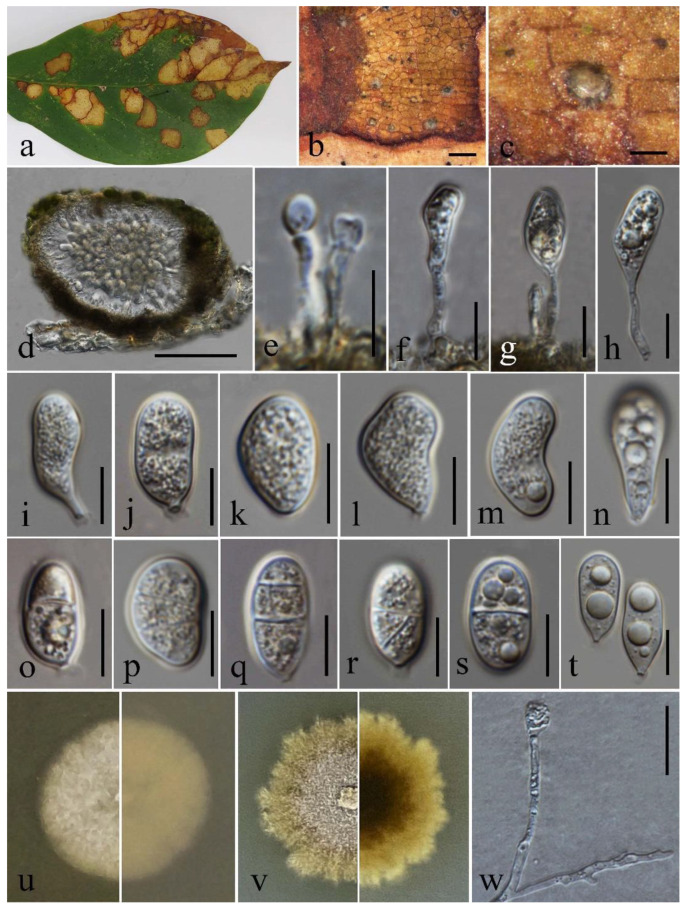
*Pseudoplagiostoma jasmini* (MFLUCC 23-0044, ex-holotype) (**a**) Leaf of *Jasminum grandiflorum* with spots. (**b**) Appearance of conidiomata on leaves. (**c**) Close up of conidioma on substrate. (**d**) Section through a conidioma. (**e**–**h**) Conidiophores, conidiogenous cells, and developing conidia. (**i**–**t**) Conidia with guttules, septa and protruding hilum. (**u**–**v**) Top (left) and reverse (right) of colonies on MEA after 7 and 14 days of incubation, respectively. (**w**) Appressorium. Scale bars: (**b**) = 1 mm, (**c**) = 200 μm, (**d**) = 100 μm, (**e**–**t**) = 10 μm, (**w**) = 20 μm.

Based on the guidelines of Chethana et al. [20], Jayawardena et al. [21], and Maharachchikumbura et al. [22] for introducing new species, we describe *P. jasmini* as a new species. Despite its support values (35% ML-IQ, 32% MP, and 0.95 PP), we establish *P. jasmini* as a new taxon, considering the formation of one or two septa in the conidia, a feature lacking in all other *Pseudoplagiostoma* species; all *Pseudoplagiostoma* spp. have aseptate conidia. Besides morphology and multigene phylogenetic analyses, we included GCPSR and PTP analyses as further evidence to support the distinct species status of *Pseudoplagiostoma jasmini* (Figure 10). 

## 5. Discussion

In pathology, appressoria are infection structures generated to invade plant tissues [1,4,69,70]. Basically, they are penetration pegs [1]. Appressoria are not solely confined to fungal pathogens. They also occur in endophytes [71,72], epiphytes [3,73,74], and saprobes [75]. In this study, we establish one new species (*Pseudoplagiostoma jasmini*), a *Ciliochorella* sp., and a new host record (*Coniella malaysiana*) that produce single-celled, irregular-shaped, hyaline appressoria. The *Ciliochorella* sp. is reported from dead leaves as a saprobe, while *P. jasmini* and *C. malaysiana* were found associated with leaf spots. In our study, pathogenicity tests were not performed. Therefore, the occurrence of appressoria in *C. malaysiana* and *P. jasmini* reveals their pathogenic and possibly endophytic nature. Certain fungi can switch their lifestyles from endophyte to saprobe and become pathogenic under suitable conditions [76]. We hypothesize that, under favorable circumstances, *C. malaysiana, P. jasmini*, and the saprobic *Ciliochorella* sp. may develop phytopathogenic traits and cause diseases. Given that appressoria are produced by fungi in various life modes, as mentioned above, it is of dire need to record their occurrences and diversity from different hosts. 

This is the first study that reports the formation of appressoria in a *Ciliochorella* sp. and a *Pseudoplagiostoma* sp., but appressoria have previously been observed in *Coniella musaiaensis* [77]. The primary function of appressoria produced by endophytes is to cross from one cell to another [3]. For saprobes to obtain their nutrients, a living host is not a requisite. Thus, the formation of an appressorium in saprobic fungi is probably a result of adaptation while in their endophytic life mode [4,78,79,80].

Species delimitation is essential to developing a proper comprehension of the biology, geography, host-fungal association, and life modes of individual fungal taxa, as well as their respective roles in the ecosystem [20]. The taxonomy of certain *Pseudoplagiostoma* species yielded low support values when constructing the phylograms (ML-IQ, MP, PP). Despite the support values for the placement of *P. jasmini*, we establish the latter as a novel taxon as there are significant differences in the conidial morphology. All *Pseudoplagiostoma* taxa, except *P. jasmini*, have aseptate conidia. Apart from *P. jasmini*, all other species of *Pseudoplagiostoma* are cryptic, sharing similar morphologies such as shape, color, and size. Therefore, coupled with morphology and phylogenetic analyses, we employed the Genealogical Concordance Phylogenetic Species Recognition Analysis (GCPSR) to infer the species boundaries in *Pseudoplagiostoma* [38]. Furthermore, we advocate the use of the coalescent-based Poisson tree processes (PTP) model to compare the inter- and intra-species genetic distances in *Pseudoplagiostoma* [41].

Many Sordariomycetes taxa are demarcated based on ITS, 28S, small subunit (18S, nuclear rDNA), *β-tub*, *tef-1α*, and *Rpb2* loci [43]. Only five *Ciliochorella* spp., but all *Pseudoplagiostoma* spp., have molecular data for one or more gene loci. A few *Ciliochorella* spp. lack sequence data for *β-tub*. The collection and examination of more *Ciliochorella* species, with the addition of more gene regions in the phylogenetic analyses, as applied in the analysis and delineation of other Sordariomycetes taxa, would lead to a better phylogenetic resolution and taxonomic placement of each species. Based on high-throughput sequencing, Baldrian et al. [81] suggested that the fungal diversity is around 6.28 million species worldwide but with only 1.08 million published species. A probable reason for the smaller number of *Ciliochorella* spp. and *Pseudoplagiostoma* spp. might be because they occur in poorly studied hosts and countries [82]. Northern Thailand is rich in fungal biodiversity [82]. Undoubtedly, further exploration of the fungal diversity in this area as well as other hotspots worldwide will reveal a higher diversity of these two and other genera [83].

## Figures and Tables

**Table 1:** GenBank accession numbers of sequences used in the phylogenetic analyses. Ex-type and reference strains are denoted with an ‘*’. Our isolates are in blue.

Species	Isolate Number	ITS	28S	*β-tub*	*Rpb2*	*tef-1α*
* Allelochaeta acuta *	CBS 144168 *	MH822973	MH823023	MH823160	N/A	N/A
* Allelochaeta acuta *	CPC 19289	MH822975	MH823025	MH823162	N/A	N/A
* Apoharknessia eucalypti *	CBS 142518 *	MG934432	MN162172	MG934505	N/A	N/A
*Apoharknessia eucalyptorum*	CBS 142519 *	KY979752	KY979807	KY979919	N/A	N/A
* Apoharknessia insueta *	CBS 111377 *	JQ706083	AY720814	N/A	N/A	MN271820
* Apoharknessia insueta *	CBS 114575	MN172402	MN172370	N/A	N/A	MN271821
*Ciliochorella castaneae*	NBRC 104545	N/A	AB433277	N/A	N/A	N/A
*Ciliochorella castaneae*	NBRC 104546	N/A	AB433278	N/A	N/A	N/A
*Ciliochorella dipterocarpi*	MFLUCC 22-0132 *	OP912991	OP912990	OQ127637	N/A	N/A
*Ciliochorella dipterocarpi*	MFLUCC 23-0023	OQ657982	OQ657981	OQ657298	N/A	N/A
*Ciliochorella * sp.	MFLUCC 23-0239	OR610581	OR610582	N/A	N/A	N/A
*Ciliochorella mangiferae*	MFLUCC 12-0310	KF827444	KF827445	KF827478	N/A	N/A
*Ciliochorella phanericola*	MFLUCC 14-0984 *	KX789680	KX789681	KX789682	N/A	N/A
*Coniella* *africana*	CBS 114133 *	AY339344	AY339293	N/A	KX833421	KX833600
*Coniella australiensis*	IMI 261318	N/A	N/A	N/A	N/A	N/A
*Coniella castaneicola*	LGZ2	MW672530	MW856810	N/A	N/A	N/A
*Coniella crousii*	NFCCI 2213	HQ264189	N/A	N/A	N/A	N/A
*Coniella diospyri*	CBS 145071 *	MK047439	MK047489	N/A	MK047543	MK047562
*Coniella diplodiella*	CBS 111858 *	AY339323	KX833335	N/A	KX833423	KX833603
*Coniella diplodiella*	CBS 111857	AY339331	AY339286	N/A	N/A	AY339357
*Coniella diplodiopsis*	CBS 590.84 *	AY339334	AY339288	N/A	N/A	AY339359
*Coniella diplodiopsis*	CBS 10923	AY339332	AY339287	N/A	KX833440	KX833624
*Coniella duckerae*	CBS 142045 *	KY924929	N/A	N/A	N/A	N/A
*Coniella erumpens*	CBS 52378 *	KX833535	KX833361	N/A	KX833446	KX833630
*Coniella eucalyptigena*	CBS 139893 *	KR476725	N/A	N/A	N/A	N/A
*Coniella eucalyptorum*	CBS 112640 *	AY339338	AY339290	N/A	KX833452	KX833637
*Coniella eucalyptorum*	CBS 114852	KX833556	KX833380	N/A	KX833464	KX833652
*Coniella ferreirense*	CBS 224.80 *	MH861257	MH873026	N/A	N/A	N/A
*Coniella fici*	MFLU 18-2578 *	MW114356	MW114417	N/A	N/A	N/A
*Coniella fici*	MFLU 18-2579	MW114357	MW114418	N/A	N/A	N/A
*Coniella fragariae*	CBS 17249 *	AY339317	AY339282	N/A	KX833472	KX833663
*Coniella fragariae*	CBS 45468	KX833571	KX833393	N/A	KX833477	KX833670
*Coniella fusiformis*	CBS 141596 *	KX833576	KX833397	N/A	KX833481	KX833674
*Coniella granati*	CBS 132860	KX833577	KX833400	N/A	KX833484	KX833677
*Coniella heterospora*	FMR: 15231	LT800501	LT800500	N/A	LT800502	LT800503
*Coniella hibisci*	CBS 109757 *	KX833589	N/A	N/A	N/A	KX833689
*Coniella javanica*	CBS 45568 *	KX833583	KX833403	N/A	KX833489	KX833683
*Coniella koreana*	CBS 14397	KX833584	AF408378	N/A	KX833490	KX833684
*Coniella lanneae*	CBS 141597 *	KX833585	KX833404	N/A	KX833491	KX833685
*Coniella limoniformis*	CBS 111021 *	KX833586	KX833405	N/A	KX833492	KX833686
*Coniella lustricola*	DAOMC 251731 *	MF631778	MF631799	N/A	MF651900	MF651899
*Coniella lustricola*	DAOMC 251734	MF631781	MF631802	N/A	N/A	N/A
*Coniella macrospora*	CBS 52473 *	KX833587	AY339292	N/A	KX833493	KX833687
*Coniella malaysiana*	CBS 141598 *	KX833588	KX833406	N/A	KX833494	KX833688
*Coniella malaysiana*	MFLUCC 23-0240	OR608286	OR608334	N/A	OR601568	OR601569
*Coniella musaiaensis*	AR3534	N/A	N/A	N/A	N/A	N/A
*Coniella nicotianae*	CBS 87572 *	KX833590	KX833407	N/A	KX833495	KX833690
*Coniella nigra*	CBS 16560 *	AY339319	KX833408	N/A	KX833496	KX833691
*Coniella obovata*	CBS 111025	AY339313	KX833409	N/A	KX833497	KX833692
*Coniella paracastaneicola*	CBS 141292 *	KX833591	KX833410	N/A	KX833498	KX833693
*Coniella peruensis*	CBS 110394 *	KJ710463	KJ710441	N/A	KX833499	KX833695
*Coniella prostata*	COAD 2597	MZ727004	MZ727000	N/A	MZ772858	MZ772860
*Coniella pseudodiospyri*	CBS 145540 *	MK876381	MK876422	N/A	MK876479	MK876493
*Coniella pseudodiospyri*	CBS 145541	MK876382	MK876423	N/A	MK876480	MK876494
*Coniella pseudogranati*	CBS 137980 *	KJ869132	N/A	N/A	N/A	N/A
*Coniella pseudokoreana*	MFLU 13-0282 *	MF190146	N/A	N/A	N/A	N/A
*Coniella pseudostraminea*	CBS 112624 *	KX833593	KX833412	N/A	KX833500	KX833696
*Coniella quercicola*	CBS 90469 *	KX833595	KX833414	N/A	KX833502	KX833698
*Coniella solicola*	CBS 76671 *	KX833597	KX833416	N/A	KX833505	KX833701
*Coniella straminea*	CBS 14922	AY339348	AY339296	N/A	KX833506	KX833704
*Coniella tibouchinae*	CBS 131595 *	JQ281774	KX833418	N/A	KX833507	JQ281778
*Coniella vitis*	JZB 3700001 *	KX890008	KX890083	N/A	N/A	KX890058
*Coniella vitis*	JZB 3700002	KX889992	KX890067	N/A	N/A	KX890042
*Coniella wangiensis*	CBS 132530 *	JX069873	JX069857	N/A	KX833509	KX833705
*Discosia ravennica*	MFLU 18-0131 *	MT376615	MT376617	MT393594	N/A	N/A
*Melanconiella hyperopta*	CBS 132231 *	MH866004	MH877448	N/A	KX833510	KX833706
*Melanconiella hyperopta*	CBS 131696	JQ926281	N/A	N/A	N/A	N/A
*Pseudoplagiostoma alsophilae*	SAUCC WZ0451 *	OP810625	OP810631	OP828586	OP828578	OP828580
*Pseudoplagiostoma alsophilae*	SAUCC WZ0152	OP810626	OP810632	OP828587	OP828579	OP828581
*Pseudoplagiostoma bambusae*	SAUCC 1206-4 *	OP810629	OP810635	OP828590	N/A	OP828584
*Pseudoplagiostoma bambusae*	SAUCC 1206-6	OP810630	OP810636	OP828591	N/A	OP828585
*Pseudoplagiostoma castaneae*	SAUCC my0162 *	MZ156982	MZ156985	MZ220325	MZ220323	MZ220321
*Pseudoplagiostoma castaneae*	SAUCC my0523	MZ156983	MZ156986	MZ220326	MZ220324	MZ220322
*Pseudoplagiostoma corymbiae*	CBS 132529 *	JX069861	JX069845	N/A	N/A	N/A
*Pseudoplagiostoma corymbiicola*	CBS 145052 *	MK047425	MK047476	MK047577	N/A	MK047558
*Pseudoplagiostoma dipterocarpi*	CMUETT57 *	KR994682	KR994683	N/A	N/A	N/A
*Pseudoplagiostoma dipterocarpicola*	MFLUCC 21-0142 *	OM228844	OM228842	OM219638	N/A	OM219629
*Pseudoplagiostoma dipterocarpicola*	MFLUCC 21-0114	OM228843	OM228841	OM219637	N/A	OM219628
*Pseudoplagiostoma eucalypti*	CBS 124807 *	GU973512	GU973606	GU973575	N/A	GU973542
*Pseudoplagiostoma eucalypti*	CBS 116382	GU973514	GU973608	GU973577	N/A	GU973544
*Pseudoplagiostoma inthanonense*	MFLUCC 23-0262 *	OR606510	OR633320	OR611920	OR611921	OR650831
*Pseudoplagiostoma jasmini * sp. nov.	MFLUCC 23-0044 *	OQ786078	OQ786079	OQ850148	N/A	OQ850145
*Pseudoplagiostoma machili*	SAUCC BW0233 *	OP810627	OP810633	OP828588	N/A	OP828582
*Pseudoplagiostoma machili*	SAUCC BW0221	OP810628	OP810634	OP828589	N/A	OP828583
*Pseudoplagiostoma mangiferae*	KUMCC 18-0197 *	MK084824	MK084825	MK084823	N/A	N/A
*Pseudoplagiostoma mangiferae*	8-1.1	MN818665	MN876855	N/A	N/A	N/A
*Pseudoplagiostoma myracrodruonis*	URM 7799 *	MG870421	MK982151	MN019566	MK977723	MK982557
*Pseudoplagiostoma myracrodruonis*	URM 8123	MK982150	MK982152	MN019567	MK977724	MK982558
*Pseudoplagiostoma oldii*	CBS 124808 *	GU973534	GU973609	GU993862	N/A	GU973564
*Pseudoplagiostoma variabile*	CBS 113067 *	GU973536	GU973611	GU993863	N/A	GU973566

N/A: Not applicable.

**Table 2:** Partition model selected for each locus for the Bayesian analyses.

	Model Selected under Akaike Information Criterion (AIC)		
Gene Region (s)	*Ciliochorella*	*Coniella*	*Pseudoplagiostoma*
ITS	HKY+G	HKY+G	GTR+G
28S	GTR+I	GTR+I+G	GTR+I+G
*β-tub*	GTR+I	N/A	HKY+I+G
*Rpb2*	N/A	GTR+I+G	GTR+G
*tef-1α*	N/A	HKY+I+G	HKY+G

N/A: Not applicable.

**Table 3:** Total number of characters, ML-IQ, and MP analysis parameters.

		*Ciliochorella*	*Coniella*	*Pseudoplagiostoma*
Number of characters in the combined alignment		2033	2975	2738
Partition of each locus		ITS: 1–516 28S: 517–1362 *β-tub*: 1363–2033	ITS: 1–580 28S: 581–1412 *Rpb2*: 1413–2175 *tef-1α*: 2176–2493	ITS: 1–557 28S: 558–1375 *β-tub*: 1376–1855 *tef-1α*: 1856–2162 *Rpb2*: 2163–2738
Number of strains used (excluding outgroups)		7 (5 species)	51 (42 species)	23 (15 species)
ML-IQ analysis parameters				
ML optimization likelihood value		−4043.048	−17,415.761	−12,799.621
ML Tree length		0.245	2.503	1.687
Distinct alignment patterns		140	916	874
Maximum parsimonious analysis parameters				
MP length: Tree #1		295	3407	2136
Constant		1747	2034	1789
Parsimony-informative		257	769	840
Parsimony-uninformative		29	172	109
Tree #1	CI	0.997	0.445	0.678
	RI	0.997	0.684	0.802
	RC	0.993	0.304	0.543
	HI	0.003	0.555	0.322

**Table 4:** Morphological comparison of *Ciliochorella* sp. (MFLUCC 23-0239) and *C. phanericola*.

		Species	
Species Characters		*Ciliochorella* sp. MFLUCC 23-0239 (This Study)	*C. phanericola* MFLUCC 14-0984 [51]
Conidiomata	Size	1000–1100 μm diam., 370–380 μm high	1000–1200 μm diam., 170–200 μm high
	Shape and colour	Semi-immersed, carbonaceous, sometimes solitary, uniloculate, black	Semi-immersed, circular areas, carbonaceous, sometimes solitary, black
Conidia	Size (μm)	11–15 × 2.4–3.8	13–15 × 2.8–3.5
	L/W	4.0	4.1
	Shape	Allantoid to sub-cylindrical, or sub-falcate to reniform, apex sometimes broadly obtuse, tapering towards a slightly curved base with an obconic basal cell, smooth	Allantoid to sub-cylindrical, smooth
	Colour	Hyaline to pale brown	Hyaline to pale brown
	Septa	1-euseptate	2-septate
	Guttules	Present	Present
Appendages		2 apical, 1 basal, tubular, filiform, flexuous; apical 12.5–18.5 μm long; basal 4–6.5 μm long	2 apical, 1 basal, tubular; apical 15–23 μm long; basal 9–11.5 μm long
Appressoria		Present	Not observed
Reported morph(s)		Asexual	Asexual
Life style(s)		Saprobic	Pathogen or saprobic on leaf
Hosts		*Jasminum* sp.	*Phanera purpurea*
Gene region(s)		ITS, 28S	ITS, 28S, *β-tub*

L/W: length-to-width ratio.

**Table 5:** Morphological comparison between our strain and the ex-type of *Coniella malaysiana*.

		Species	
Species Characters		*C. malaysiana* MFLUCC 23-0240 (This Study)	*C. malaysiana* CBS 141598 [55]
Conidiomata	Size	135–140 μm diam., 100–130 μm high	550 μm diam.
	Shape and color	Semi-immersed, sometimes erumpent, solitary, scattered or gregarious, uniloculate, globose to subglobose, black	Immersed or superficial, globose to depressed, initially hyaline, becoming olivaceous to black with age
Conidia	Size (μm)	8.2–13.1 × 4–5	8.5–11 × 3.5–4.5
	L/W	2.6	2.5
	Shape	Smooth, thick-walled, fusiform to truncate to sub-ellipsoidal, sometimes obovoid, tapering towards a slightly curved apex and base, wider in the middle	Thick-walled, fusoid to ellipsoid, inequilateral, apex acutely rounded, widest in the middle, tapering to a truncate base
	Color	Hyaline when immature, becoming pale to dark brown upon maturation	Hyaline to pale brown
	Septa	Aseptate	Aseptate
	Guttules	Present	Present
Appressoria		Present	Not observed
Reported morph(s)		Asexual	Asexual
Life style		Associated with leaf spots	Plant pathogenic
Hosts		*Jasminum* sp.	*Corymbia torelliana*
Gene region(s)		ITS, 28S, *Rpb2*, *tef-1α*	ITS, 28S, *Rpb2*, *tef-1α*

L/W: Length to width ratio.

**Table 6:** Morphological comparison of *Pseudoplagiostoma jasmini* with *P. dipterocarpicola*.

		Species	
Species Characters		*P. jasmini* MFLUCC 23-0044 (This Study)	*P. dipterocarpicola* MFLUCC 21-0142 [67]
Conidiomata	Size	150–230 μm diam., 140–200 μm high	113–288 μm diam., 63–153 μm high
	Shape and color	Pycnidial, semi-immersed, globose to subglobose, pale brown, surrounded with black margin	Pycnidial with pale yellow cylindrical strips of exuding conidia, subglobose, subcuticular to epidermal, unilocular, irregularly breaking through plant tissue at the center, medium to dark brown
Conidia	Size (μm)	14–22 × 6.5–11	9–22 × 4–7.5
	L/W	2.0	2.7
	Shape	Cylindrical to truncate or ellipsoidal, elongated, reniform, pyriform or obovoid, apex broadly obtuse, tapering towards slightly curved base	Ellipsoidal to elongated, apex broadly obtuse, straight, or slightly curved at base, often slightly narrow at middle, base tapering to flat protruding scar
	Color	Hyaline	Hyaline
	Septa	0–2-septate	Aseptate
	Guttules	Present	Present
	Conidial wall (μm)	Smooth, 0.6–1.3	Smooth, 0.5–1.5
Hilum		Mostly present and prominent	Present or absent
Appressoria		Present	Not observed
Reported morph(s)		Asexual	Asexual
Life style		Associated with leaf spots	Associated with twigs and fruits
Hosts		*Jasminum grandiflorum*	*Dipterocarpus* sp.
Gene region(s)		ITS, 28S, *β-tub*, *tef-1α*	ITS, 28S, *β-tub*, *tef-1α*

L/W: Length-to-width ratio.

**Table 7:** Genetic distance (%) between *Pseudoplagiostoma* species (grouped according to PTP results) in the concatenated ITS, 28S, *β-tub*, and *tef-1α* genetic markers.

	Group 1 (%)	Group 2 (%)	Group 3 (%)	Group 4 (%)
Group 1: *P. machili*	N/A	5.67	11.0	12.3
Group 2: *P. alsophilae*	5.67	N/A	10.1	11.8
Group 3: *P. dipterocarpicola*	11.0	10.1	N/A	9.11
Group 4: *P. jasmini*	12.3	11.8	9.11	N/A

N/A: not applicable.

## Data Availability

All data generated or analyzed during this study are included in this published article.
